# Lipemic Plasma Identified Blood Donors: Triglyceride Variability and Exploratory Machine Learning Analysis

**DOI:** 10.3390/medsci14010140

**Published:** 2026-03-17

**Authors:** Sirinya Sitthirak, Sodsai Narkpetch, Rujira Nonsa-ard, Manit Nuinoon, Poonsup Sripara, Krittamate Saisuwan, Saengrawee Thammawithan, Yanisa Rattanapan

**Affiliations:** 1 Department of Medical Technology, School of Allied Health Sciences, Walailak University, Nakhon Si Thammarat 80160, Thailand; sirinya.sit@wu.ac.th (S.S.); manit.nu@wu.ac.th (M.N.); 2 Research Excellence Center for Innovation and Health Products (RECIHP), Walailak University, Nakhon Si Thammarat 80160, Thailand; 3 Blood Bank, Maharaj Nakhon Si Thammarat Hospital, Nakhon Si Thammarat 80000, Thailand; sodsai_nark@yahoo.com; 4 Faculty of Public Health, Mahasarakham University, Mahasarakham 44150, Thailand; rujira.n@msu.ac.th; 5 Hematology and Transfusion Science Research Center, Walailak University, Nakhon Si Thammarat 80160, Thailand; 6 Blood Transfusion Center, Faculty of Medicine, Khon Kaen University, 123 Mittraparp Highway, Muang District, Khon Kaen 40002, Thailand; poonsr@kku.ac.th; 7 Department of Immunopharmacology, Graduate School of Medicine, Kyoto University, Kyoto 606-8507, Japan; saisuwan.krittamate.2y@kyoto-u.ac.jp; 8 Cholangiocarcinoma Research Institute, Khon Kaen University, Khon Kaen 40002, Thailand; saenth@kku.ac.th

**Keywords:** blood donors, triglycerides, cardiovascular risk, dyslipidemia, machine learning

## Abstract

Background/Objectives: Early detection of cardiometabolic irregularities is crucial for averting cardiovascular illness; however, demographic cohorts that consistently engage with healthcare systems like habitual blood donors are inadequately leveraged for metabolic monitoring. Methods: This study performed lipid profiling and cardiovascular risk assessment among blood donors identified with visually lipemic plasma during routine screening, in order to explore metabolic variability within this selected donor subgroup. Of 13,818 screened donors, 160 with lipemic plasma were included, and multivariable and machine-learning analyses were restricted to 90 donors with complete clinical data. Results: We observed substantial variability in triglyceride levels, with males displaying higher and more dispersed values. Correlation analysis indicated that triglycerides were associated with BMI and composite cardiovascular risk metrics, while age was the strongest contributor to the calculated 10-year cardiovascular risk score. Using a Random Forest classifier, elevated triglyceride levels were predicted with an AUC of 0.86; however, given the limited sample size, this analysis should be interpreted as exploratory and proof-of-concept in nature. Conclusions: In this selected subgroup of donors with lipemic plasma, clinically relevant hypertriglyceridemia was frequently observed. These findings suggest that routine donor data may provide opportunities for targeted metabolic monitoring, although the results cannot be generalized to the broader blood donor population. Further studies in larger and more representative cohorts are warranted.

## 1. Introduction

Cardiovascular disease (CVD) continues to be the predominant cause of mortality globally, responsible for almost one-third of all fatalities, mostly influenced by preventable metabolic risk factors including dyslipidemia, obesity, and hypertension [1]. In Southeast Asia, especially Thailand, the incidence of premature cardiovascular disease is escalating due to lifestyle changes and a growing prevalence of metabolic disorders, highlighting the necessity for early detection and focused prevention [2,3,4]. Triglyceride and cholesterol dysregulation are pivotal in cardiometabolic pathways and are incorporated into primary cardiovascular risk algorithms that inform worldwide prevention strategies [5].

Regular blood donors constitute a significant but under examined demographic for monitoring cardiometabolic risk. Despite the conventional perception that blood donors are healthier than the general populace due to rigorous eligibility standards a phenomenon termed the “healthy donor effect” recent studies reveal a rising incidence of metabolic disorders, including hypertriglyceridemia and obesity, even among regular donors [6,7,8]. While donor cohorts have been proposed as potential platforms for health monitoring, most available evidence remains limited and context-specific [9,10].

Notwithstanding this potential, limited research in Thailand has systematically assessed lipid profiles or cardiovascular risk among blood donors, particularly in high-burden areas such as Southern Thailand. The Blood Bank Unit of Maharaj Nakhon Si Thammarat Hospital serves as a major provincial transfusion facility with a stable base of repeat donors, providing an opportunity to explore metabolic variability within this clinical setting.

Importantly, the present study focuses specifically on donors identified with visually lipemic plasma during routine screening, rather than representing the general donor population. By examining triglyceride variability and associated cardiometabolic indicators within this selected subgroup, this study aims to generate exploratory insights into metabolic patterns observed in routine blood bank practice.

This study conducted lipid profiling and cardiovascular risk evaluation among donors with visually lipemic plasma at Maharaj Nakhon Si Thammarat Hospital. We investigated relationships between lipid indicators and calculated risk scores, assessed sex-specific variation in triglyceride levels, and evaluated the exploratory performance of machine-learning models for identifying elevated triglyceride concentrations. Rather than providing population-level estimates, this analysis seeks to characterize metabolic variability within a selected donor subgroup and to assess the feasibility of data-driven approaches in this context.

### 1.1. Study Population and Sample Collection

This laboratory-based cross-sectional study was conducted at the Blood Bank Laboratory of Maharaj Nakhon Si Thammarat Hospital, Thailand, over a one-year period from November 2022 to November 2023. During this period, 13,818 blood donors were screened as part of routine pre-donation eligibility assessment. Under Thai national blood donation criteria, individuals with known diabetes mellitus requiring ongoing medical treatment are not eligible for donation. Consistent with this screening policy, no donors with documented diabetes mellitus were included in the present analytic cohort.

Among the 13,818 screened donors, 160 were identified as having visually confirmed lipemic plasma during routine laboratory inspection and met the predefined inclusion criteria for this study. Of these 160 lipemic donors, 90 (56.3%) had complete demographic, anthropometric, biochemical, and cardiovascular risk data and were included in the final multivariable and machine-learning analyses. The restriction to complete-case records was applied to ensure analytical consistency and model stability.

Blood samples used for biochemical analysis were collected during routine pre-donation assessment, prior to completion of the blood donation procedure. Eligible participants were voluntary blood donors with sufficient blood volume for laboratory analysis. Samples exhibiting hemolysis or icteric plasma were excluded to avoid analytical interference.

Ethical approval was obtained from the Walailak University Ethics Committee for Human Research (WUEC-22-334-01), and the study was conducted in accordance with the Declaration of Helsinki. Written informed consent was obtained from all participants prior to inclusion. This study therefore reflects a selected subgroup enriched for elevated triglyceride levels rather than the overall donor population.

### 1.2. Data Acquisition

Data were obtained from donor application forms and laboratory records, encompassing demographic details such as sex and age, anthropometric measurements including weight and height (normalize for calculating body mass index, BMI), physiological parameters such as pulse rate, and biochemical data comprising cholesterol (CHO) and triglyceride (TG) levels. Supplementary information collected encompassed smoking normalize within the preceding 24 h, diabetes status, and the quantity of donations. Cardiovascular risk was evaluated utilising 10-year CVD risk scores and the Thai CV Risk Score, with relative risk values computed based on age- and sex-matched controls. Triglyceride measurements were obtained under routine non-fasting conditions, as donors were not required to fast prior to donation. Triglyceride measurements were obtained under routine non-fasting conditions, as donors were not required to fast prior to donation. Triglyceride categories were defined using conventional clinical cut-offs (normal <150 mg/dL, borderline 150–199 mg/dL, high 200–499 mg/dL, very high ≥500 mg/dL) to facilitate comparability with widely used guideline-based classifications; however, we acknowledge that applying fasting thresholds to non-fasting measurements may introduce category misclassification.

### 1.3. Management of Blood Samples and Laboratory Examination

Whole blood specimens were collected during routine pre-donation assessment, prior to completion of the blood donation procedure. Samples were drawn into 6 mL CAT Serum Clot Activator tubes and processed promptly according to standard laboratory protocols to minimize pre-analytical variability.

Serum triglyceride levels were measured using an automated biochemical analyzer based on standard enzymatic methods routinely employed in the hospital laboratory. Plasma lipemia was assessed visually during routine laboratory inspection. Donors exhibiting visibly turbid or milky plasma were classified as having lipemic plasma and were included in the study cohort.

### 1.4. Cardiovascular Risk Scoring

The validated Thai 10-year cardiovascular disease (CVD) risk score, designed for the Thai population, was normalized for cardiovascular risk calculation, incorporating regionally pertinent epidemiological factors. This risk prediction model assesses the likelihood of experiencing fatal or non-fatal cardiovascular events within a decade, utilising commonly accessible clinical and demographic data.

The 10-year CVD risk for each participant was calculated using age, sex, systolic blood pressure, total cholesterol, smoking status, and diabetes status, in accordance with the Thai CVD Risk Score algorithm. All required input variables were obtained from pre-donation health assessment records at the Blood Bank Unit. Risk scores were expressed as continuous percentages and were analyzed as continuous variables for both descriptive and inferential statistical analyses.

Alongside the definitive 10-year risk assessment, a relative cardiovascular risk index was calculated for each donor to facilitate age-specific risk normalization, enabling comparisons among individuals from diverse age cohorts. This relative risk metric indicates an individual’s expected risk compared to a reference subject of identical age and sex without significant cardiovascular risk factors, thereby offering further insights into cardiovascular susceptibility beyond absolute risk levels.

The computed risk scores were regarded as continuous variables for correlation analysis with lipid parameters and anthropometric measurements and were also employed to delineate the total cardiovascular risk distribution within the donor group. All cardiovascular risk assessments were executed within the computational analytic framework to guarantee reproducibility and uniformity across analyses.

### 1.5. Descriptive and Statistical Examination

Descriptive statistics were produced to delineate the metabolic and demographic characteristics of the donor population. Continuous data, such as total cholesterol, triglycerides, body mass index, blood pressure, pulse rate, and cardiovascular risk scores, were summarized utilizing means, standard deviations, medians, and interquartile ranges. Categorical factors, including sex, smoking status, diabetes history, blood group, and donor type, were summarized through frequencies and proportions. All analyses were conducted on records with complete data, adhering to a uniform preprocessing pipeline executed in Python.

### 1.6. Analysis of Correlation

**Figure 1:**
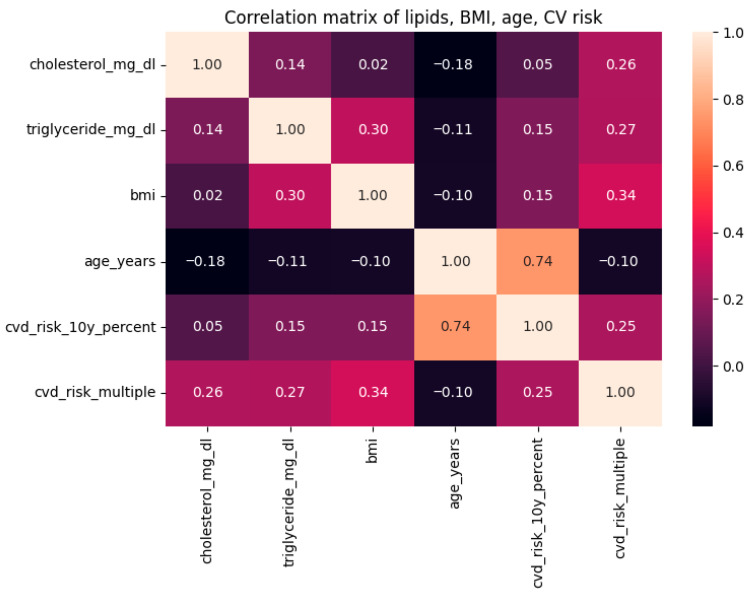
Correlation heatmap depicting relationships among lipid and cardiovascular factors. The heatmap illustrates Pearson connection coefficients among blood total cholesterol, triglycerides, body mass index (BMI), age, a 10-year Thai cardiovascular disease (CVD) risk score, and the relative cardiovascular risk index. Positive correlations are represented by increasingly darker shades, whereas lighter hues denote weaker or insignificant connections. The most robust link was noted between age and the 10-year cardiovascular disease risk score (r = 0.74), although triglycerides demonstrated a modest correlation with BMI (r = 0.30) and lesser relationships with cardiovascular risk metrics. This picture encapsulates the fundamental linear relationships that underpin later regression and predictive modeling techniques.

Pearson correlation coefficients were calculated to examine associations between lipid parameters and cardiovascular risk factors, including total cholesterol, triglycerides, BMI, age, the Thai 10-year CVD risk score, and the relative CVD risk index. The correlation matrix was visualized using a heatmap (Figure 1). To account for potential non-normality and right-skewness in triglyceride distribution, Spearman’s rank correlation (ρ) was also computed in parallel. Together, these analyses enabled assessment of both linear (Pearson’s r) and monotonic (Spearman’s ρ) relationships among metabolic and cardiovascular variables.

### 1.7. Comparative Analysis of Groups

Triglyceride distributions were compared between male and female donors. Given the right-skewed distribution of triglyceride levels, differences between groups were assessed using the Mann–Whitney U test. Boxplots were generated to illustrate group-level differences, variability, and outliers in triglyceride concentrations. No donors in the complete-case dataset had documented diabetes mellitus; therefore, comparison by diabetes status was not performed.

### 1.8. Logistic Regression Analysis

A multivariable logistic regression model was employed to ascertain independent predictors of increased triglycerides, defined as a serum triglyceride concentration of ≥200 mg/dL. The predictor variables comprised age, BMI, smoking status, diabetes status, and sex. Variables exhibiting no variance were automatically eliminated to prevent model instability. Maximum likelihood estimation was conducted utilizing the statsmodels package, with regression coefficients, standard errors, and significant values documented. Model diagnostics and statistical results are provided in the computational notebook, illustrating the parameters most significantly correlated with triglyceride rise in this donor cohort. Model assumptions were evaluated prior to interpretation. Linearity of the logit for continuous predictors (age and BMI) was assessed using the Box–Tidwell approach. Multicollinearity was examined using variance inflation factors (VIF). Model goodness-of-fit was evaluated using the Hosmer–Lemeshow test in addition to the likelihood ratio test.

### 1.9. Predictive Model Utilizing Machine Learning for Triglyceride Levels

A Random Forest classifier was developed to explore the predictive potential of routinely collected demographic and clinical donor features. The dataset was partitioned into training and test sets using a stratified 70:30 split to preserve class distribution. The model was configured with 300 trees and class-balanced weighting to mitigate class imbalance. Model performance was evaluated using precision, recall, F1-score, and the area under the receiver operating characteristic curve (AUC).

The initial stratified split yielded an AUC of 0.86. However, given the limited sample size (n = 90) and the relatively small independent test set (approximately 27 donors), performance estimates may be unstable and sensitive to random partitioning. Accordingly, this modeling approach should be interpreted as exploratory and proof-of-concept rather than definitive predictive modeling.

No extensive hyperparameter tuning, cross-validation, or bootstrap resampling was performed due to the limited sample size, as such procedures may increase the risk of overfitting in small datasets. The model was therefore implemented using standard, conservative parameters to provide an initial feasibility assessment.

### 1.10. Software

All data cleansing, statistical analyses, visualizations, and machine learning modeling were conducted using Python 3.10 in Google Colab. The core libraries encompassed pandas for data manipulation, NumPy for numerical calculation, seaborn and matplotlib for visualization, statsmodels for regression modeling, and scikit-learn for machine learning implementation. The entire computational workflow, encompassing preprocessing procedures, produced figures, and saved outputs, is meticulously documented in the corresponding analysis notebook. Methodological frameworks for logistic regression and Random Forest modeling followed established statistical and machine-learning references.

## 2. Results

### 2.1. Baseline Characteristics of the Study Population

Ninety routine blood donors were incorporated into the analysis shown in Table 1. The average age of the participants was 40.3 years (SD 10.7, range 19–57). The mean body mass index (BMI) was 26.26 kg/m^2^ (SD 4.81), with a range from 17.65 to 46.09 kg/m^2^. Serum lipid profiling revealed a mean total cholesterol content of 212.9 mg/dL (SD 38.5), with levels ranging from 135 to 299 mg/dL. Triglyceride levels exhibited significant variability, with a mean of 223.8 mg/dL (SD 164.7) and a broad range from 57 to 1154 mg/dL.

The median 10-year Thai cardiovascular disease (CVD) risk score was 2.62%, whereas the mean risk was 3.62% (SD 2.98), with individual scores varying from 0.52% to 15.67%. The data reveal a primarily low-risk group at baseline; however, significant variability in lipid readings, especially triglycerides, was seen despite donors satisfying national fitness standards for blood donation.

### 2.2. Distribution of Triglyceride Categories

The triglyceride levels among the 90 blood donors exhibited significant variability (Table 2). Approximately one-third of the individuals (33.3%, n = 30) had triglyceride levels within the normal range (<150 mg/dL). Borderline increase (150–199 mg/dL) was noted in 23 donors (25.6%), whereas high triglyceride levels (200–499 mg/dL) were the biggest segment, with 32 persons (35.6%). Significantly, five donors (5.6%) demonstrated markedly elevated triglyceride levels (≥500 mg/dL), with several instances above 1000 mg/dL. These data indicate that, despite being a clinically prescreened population often regarded as healthy, over 40% of donors had triglyceride levels beyond the optimal range, with a minor yet significant subset presenting severe hypertriglyceridemia. This trend highlights the metabolic heterogeneity among regular blood donors and indicates that increased triglycerides may be more prevalent in this demographic than previously anticipated.

### 2.3. Correlation Heatmap Among Lipid and Cardiovascular Variables

The correlation heatmap demonstrated clear relationship patterns across lipid markers, anthropometric measurements, and cardiovascular risk factors (Figure 1). Triglyceride levels had a moderate positive association with BMI (r = 0.30) and a weaker association with the 10-year cardiovascular risk score (r = 0.15). Total cholesterol exhibited a limited association with triglycerides (r = 0.14) and negligible relationships with other clinical factors.

Age demonstrated the most significant correlation within the dataset, exhibiting a robust positive association with the 10-year cardiovascular disease (CVD) risk score (r = 0.74), reflecting the structural emphasis of age in the Thai cardiovascular risk algorithm. BMI showed moderate relationships with cardiovascular risk indicators (r = 0.25–0.30). No substantial negative associations were observed among the analyzed variables.

Overall, the heatmap indicates that although triglycerides and BMI contribute modestly to variability in estimated cardiovascular risk, age remains the dominant determinant in this donor subgroup. These patterns informed the subsequent regression and predictive modeling analyses by clarifying the underlying linear relationships among variables. Spearman’s rank correlation analysis yielded comparable patterns, confirming a moderate monotonic association between triglycerides and BMI (ρ = 0.42) and a strong association between age and the 10-year CVD risk score (ρ = 0.85), thereby supporting the robustness of these findings despite the right-skewed distribution of triglycerides.

### 2.4. Sex-Specific Differences in Triglyceride Concentrations

**Figure 2:**
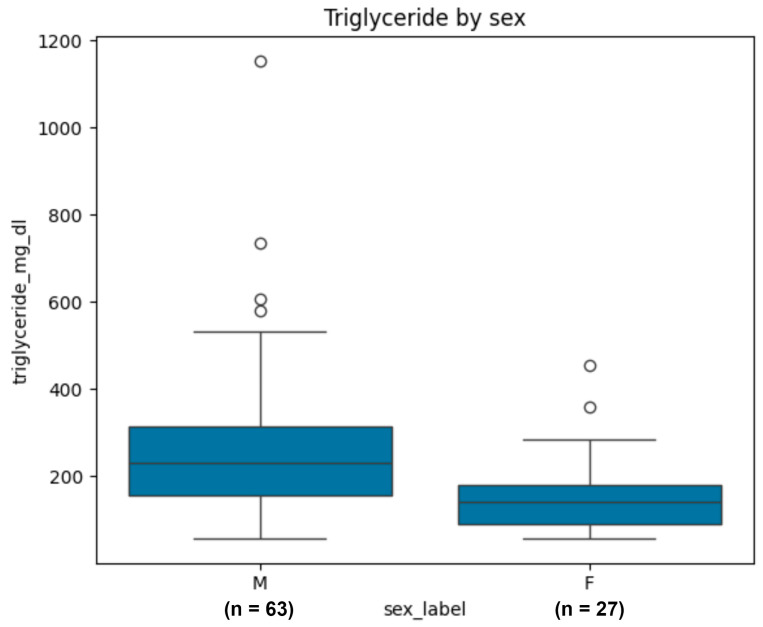
Boxplots of triglyceride concentrations by sex among complete-case donors (n = 90). Triglyceride levels (mg/dL) are shown separately for male (n = 63) and female (n = 27) donors. The boxplots represent the median (central line), interquartile range (IQR; box), and minimum–maximum values excluding outliers (whiskers). Individual outliers are displayed as points.

Triglyceride levels shown significant variation across male and female blood donors shown in Figure 2. The boxplot indicated that males displayed significantly elevated median triglyceride levels relative to females and exhibited a wider overall dispersion. Extreme values were primarily noted in male donors, with some people exhibiting triglyceride levels exceeding 500 mg/dL, and in certain instances, approaching 1000 mg/dL. Conversely, female donors exhibited a more limited range, characterized by a lower central tendency and an absence of extremely elevated triglyceride levels. This gender-specific gap underscores a significant metabolic variation among a preselected donor cohort. The results align with established physiological and hormonal effects on lipid metabolism, reinforcing the necessity of include sex as a crucial predictor in future regression and machine-learning investigations.

### 2.5. Association Between Triglyceride Categories, Sex, and Donation Type

The distribution of triglyceride categories varied significantly by gender (Table 3). Over fifty percent of female donors exhibited triglyceride levels within the normal range (<150 mg/dL), while only one in five male donors displayed normal levels. Elevated triglyceride levels (200–499 mg/dL) were primarily noted in males, who comprised approximately fifty percent of all patients within this classification. All instances of significantly elevated triglycerides (≥500 mg/dL) were solely found in male donors, highlighting the marked gender gap in lipid metabolism within this prescreened donor cohort.

### 2.6. Determinants of High Triglyceride Concentration in Multivariable Analysis

Multivariable logistic regression was conducted to evaluate factors independently associated with elevated triglyceride levels (≥200 mg/dL) (Table 4). After adjustment for age, BMI, smoking status, and sex, two variables remained statistically significant. Male sex was independently associated with higher odds of elevated triglyceride levels (β = 2.26, OR = 9.58, *p* = 0.001), indicating that male donors had substantially greater odds of exceeding the triglyceride threshold compared with female donors. BMI was also independently associated with elevated triglycerides (β = 0.13, OR = 1.13, *p* = 0.029), suggesting that each unit increase in BMI was associated with a higher likelihood of triglyceride levels ≥ 200 mg/dL.

Age was not statistically significant in the adjusted model (*p* = 0.157), although a modest negative trend was observed. Smoking within the preceding 24 h was not significantly associated with elevated triglycerides (*p* = 0.875). No donors in the complete-case dataset had documented diabetes mellitus; therefore, diabetes status was not included in the final model.

Overall model fit was assessed using the likelihood ratio test, which indicated a statistically significant improvement compared with the null model (*p* < 0.001). These findings reinforce the observed sex-specific pattern in triglyceride distribution and highlight BMI as an important factor associated with triglyceride variability within this selected donor subgroup.

### 2.7. Machine-Learning Performance for Predicting High Triglyceride Levels

**Figure 3:**
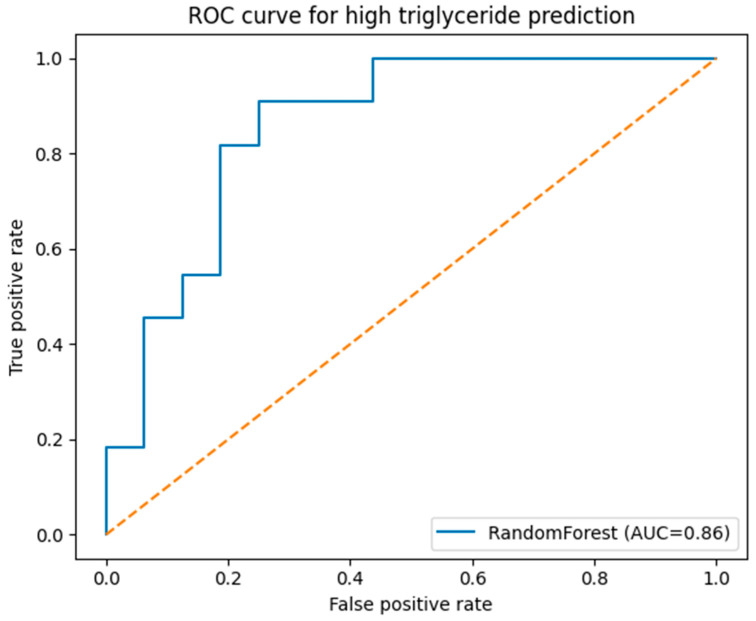
Receiver operating characteristic (ROC) curve of the Random Forest model for prediction of high triglyceride levels (≥200 mg/dL). The ROC curve illustrates the discriminatory performance of the Random Forest classifier in identifying donors with high triglyceride levels (≥200 mg/dL) using routinely collected demographic and clinical variables. The model was trained using a stratified 70:30 train–test split on the complete-case dataset (n = 90), and performance metrics were calculated on the independent test set. The area under the curve (AUC) was 0.86, indicating good predictive accuracy. The diagonal reference line represents random classification performance.

The exploratory machine-learning analysis demonstrated moderate discriminatory capacity in identifying donors with elevated triglyceride levels (≥200 mg/dL). Using routinely collected demographic and clinical variables; the Random Forest classifier achieved an area under the receiver operating characteristic curve (AUC) of 0.86 in the independent test set (Figure 3, Table 5). The ROC curve deviated from the reference diagonal, suggesting discrimination above random classification.

However, given the limited overall sample size (n = 90) and the relatively small test set (approximately 27 donors), performance metrics should be interpreted with caution. Estimates derived from a single 70:30 split may be sensitive to random partitioning and therefore potentially unstable. No cross-validation or bootstrap-based resampling was performed, and hyperparameter tuning was intentionally limited to reduce the risk of overfitting in this small dataset.

Accordingly, these findings should be regarded as exploratory and proof-of-concept in nature. External validation in larger and more representative donor cohorts is required before considering any operational or clinical application of predictive modeling in this context.

## 3. Discussion

This study demonstrates that clinically significant metabolic anomalies exist among routine blood donors, a demographic generally presumed to be healthier than the broader population due to rigorous pre-donation eligibility assessments. Contrary to this expectation, over 40% of donors demonstrated borderline to elevated triglyceride levels, with a fraction exhibiting extremely high values over 500 mg/dL. These findings align with research indicating that dyslipidemia frequently goes undetected in adults, including those lacking evident cardiometabolic symptoms [11,12]. However, because this cohort was enriched for lipemia, these prevalence estimates should not be extrapolated to all blood donors.

This study reveals a significant gender difference in triglyceride levels. Male donors exhibited significantly elevated median triglyceride levels and increased variability, encompassing all instances of markedly high triglycerides. This corresponds with recognized biological and behavioral factors influencing lipid metabolism, since males generally have stronger atherogenic lipid profiles and increased hepatic VLDL production [13,14,15]. In multivariable analysis, male sex was independently associated with, rather than causally linked to, elevated triglycerides. These findings support the importance of considering sex-specific patterns when interpreting lipid variability in similar clinical contexts.

Body mass index also proved to be a significant factor in elevated triglycerides. This study’s positive correlation is substantiated by molecular connections among obesity, insulin resistance, and disrupted lipoprotein metabolism [16,17]. While age significantly affected cardiovascular risk scores, as anticipated from the Thai 10-year CVD risk algorithm, it could not independently forecast increased triglycerides, indicating that hypertriglyceridemia may occur across a wide age spectrum among donor communities.

The exploratory machine-learning analysis demonstrated that routine pre-donation variables could achieve discrimination of elevated triglyceride status, with an AUC of 0.86. Given the limited sample size and the small independent test set, this result should be interpreted as proof-of-concept rather than definitive predictive performance. Machine-learning approaches have increasingly been applied in cardiovascular risk modeling to detect nonlinear interactions [18,19]; however, external validation in larger and more representative cohorts is necessary before clinical implementation can be considered.

The finding that single-donor platelet (SDP) donors displayed a greater prevalence of increased triglycerides than whole-blood donors necessitate additional research. Although the mechanism is not fully understood, previous research suggests that apheresis donors may exhibit distinct behavioral or metabolic traits compared to whole-blood donors [20]. Extensive, multi-center investigations may elucidate if the method of donation, its frequency, or other confounding factors contribute to this trend.

From a practical perspective, this study raises the possibility that blood bank data may provide insight into metabolic variability within specific high-risk donor subgroups. However, the present findings do not justify population-level screening recommendations. Rather, they suggest that further investigation into targeted metabolic assessment strategies within donor settings may be warranted, particularly in regions experiencing rising cardiometabolic risk [21], utilizing donor programs may provide a scalable, cost-effective solution for identifying undiagnosed dyslipidemia. Integrating basic digital risk indicators or predictive technologies into donation processes may facilitate prompt referrals for subsequent evaluation.

This study possesses multiple limitations. Given the cross-sectional and observational design, causal inferences cannot be established. The reported associations reflect relationships observed within a selected lipemic donor subgroup and should not be interpreted as evidence of causal effects. Reverse causation and residual confounding cannot be excluded.

The sample size was modest and derived from a single center, limiting generalizability. Triglyceride levels were obtained under non-fasting conditions, which may have influenced classification; therefore, triglyceride category interpretation should be approached with caution. In addition, comprehensive lipid fractions (HDL-C and LDL-C), glucose measurements, and markers of insulin resistance were not consistently available, thereby limiting full cardiometabolic characterization. Furthermore, the study specifically focused on donors with visibly lipemic plasma and therefore does not represent the broader blood donor population. Future investigations should incorporate longitudinal follow-up, multi-center cohorts, and more comprehensive metabolic profiling.

Notwithstanding these constraints, the study offers preliminary insights into triglyceride variability among donors exhibiting lipemic plasma under standard treatment. The amalgamation of traditional statistical modelling with machine-learning techniques provides complementary insights into metabolic correlations within this specific clinical situation. The results reveal clinically significant hypertriglyceridemia, including severe increases (≥500 mg/dL), among a cohort of donors often considered low risk, emphasising the necessity for careful further exploration of focused risk assessment methodologies.

## 4. Conclusions

This study reveals that clinically significant hypertriglyceridemia was commonly found in a specific subgroup of blood donors with visibly lipemic plasma, rather than in the overall donor population. Male sex and increased BMI were independently correlated with heightened triglyceride levels in multivariable analysis, whereas the machine-learning model demonstrated preliminary proof-of-concept predictive efficacy based on routinely gathered pre-donation data.

These results must be regarded with caution due to the chosen cohort and restricted sample size. Nonetheless, the findings indicate that regularly gathered donor data may provide avenues for focused metabolic evaluation within particular high-risk groupings, contingent upon validation in larger and more representative investigations.

## Figures and Tables

**Table 1:** Baseline characteristics of complete-case blood donors (n = 90). Continuous variables are presented as mean ± standard deviation (SD) and additionally as median with interquartile range (Q1–Q3), minimum, and maximum values to reflect data distribution. BMI, body mass index; CVD, cardiovascular disease. The 10-year CVD risk was calculated using the validated Thai CVD Risk Score algorithm. Analyses were performed using complete-case data only.

Variable	Mean ± SD	Min	Q1	Median	Q3	Max
Cholesterol (mg/dL)	212.88 ± 38.47	135	184.5	208	241.5	299
Triglyceride (mg/dL)	223.81 ± 164.71	57	111	185.5	263	1154
BMI (kg/m^2^)	26.26 ± 4.81	17.65	23.14	25.22	29.11	46.09
Age (years)	40.34 ± 10.68	19	32.25	40.5	49	57
10-year CVD risk (%)	3.62 ± 2.98	0.52	1.39	2.62	5.08	15.67

**Table 2:** Distribution of triglyceride (TG) categories among complete-case donors (n = 90). Triglyceride categories were defined according to conventional clinical cut-offs: normal (<150 mg/dL), borderline high (150–199 mg/dL), high (200–499 mg/dL), and very high (≥500 mg/dL). Values are presented as absolute counts (n) and percentages (%).

TG Category	n	%
Normal (<150)	30	33.30%
Borderline (150–199)	23	25.60%
High (200–499)	32	35.60%
Very high (≥500)	5	5.60%

**Table 3:** Distribution of triglyceride categories by sex among complete-case donors (n = 90). Values are presented as n (%) within each sex group. TG categories were defined as described in Table 2.

Sex	Normal	Borderline	High	Very High
Male	14 (22%)	13 (21%)	30 (48%)	6 (9%)
Female	14 (53%)	9 (34%)	3 (12%)	0 (0%)

**Table 4:** Multivariable logistic regression analysis of factors associated with elevated triglyceride levels (≥200 mg/dL) among complete-case donors (n = 90). The outcome variable was high triglyceride status (≥200 mg/dL). Sex was coded as a binary variable with female as the reference category; therefore, only the coefficient for male sex is displayed. Coef = logistic regression coefficient (log-odds); OR = odds ratio; CI = confidence interval. Estimates were derived using maximum likelihood estimation, and *p*-values were calculated using the Wald test. Variables with zero variance (e.g., diabetes status) were not included in the final model.

Predictor	Coef	OR	95% CI	*p*-Value
Male sex	2.259	9.58	2.67–34.36	0.001
BMI	0.1266	1.13	1.01–1.27	0.029
Age (years)	−0.0347	0.97	0.92–1.01	0.157
Smoking < 24 h	0.131	1.14	0.22–5.81	0.875

**Table 5:** Random Forest model performance. Performance metrics of the Random Forest classifier for predicting high triglyceride levels (≥200 mg/dL). The dataset (n = 90) was divided into training and testing sets using a stratified 70:30 split. Performance metrics were calculated on the independent test set. Precision, recall, and F1-score are reported separately for Class 0 (normal TG < 200 mg/dL) and Class 1 (high TG ≥ 200 mg/dL). AUC, area under the receiver operating characteristic curve.

Metric	Class 0 (Normal TG)	Class 1 (High TG)
Precision	0.867	0.75
Recall	0.812	0.818
F1-score	0.839	0.783
Overall Accuracy	0.815	NA
AUC	0.858	NA

## Data Availability

The original contributions presented in this study are included in the article. Further inquiries can be directed to the corresponding author(s).

## Abbreviations

The following abbreviations are used in this manuscript:

TG	Triglycerides
CHO	Total cholesterol
BMI	Body mass index
CVD	Cardiovascular disease
CV risk	Cardiovascular risk
SDP	Single-donor platelet
ROC	Receiver operating characteristic
AUC	Area under the curve
VLDL	Very-low-density lipoprotein
HDL-C	High-density lipoprotein cholesterol
LDL-C	Low-density lipoprotein cholesterol
DM	Diabetes mellitus
BP	Blood pressure
IQR	Interquartile range
OR	Odds ratio (logistic regression)
ML	Machine learning
RF	Random Forest
CI	Confidence interval
SD	Standard deviation
